# Phenotypic and Genotypic Investigation of Two Representative Strains of Microbacterium Species Isolated From Micro-Filtered Milk: Growth Capacity and Spoilage-Potential Assessment

**DOI:** 10.3389/fmicb.2020.554178

**Published:** 2020-10-22

**Authors:** Paolo Bellassi, Fabrizio Cappa, Alessandra Fontana, Lorenzo Morelli

**Affiliations:** ^1^Department for Sustainable Food Process (DiSTAS), Università Cattolica del Sacro Cuore, Piacenza, Italy; ^2^Biotechnology Research Centre (CRB), Cremona, Italy

**Keywords:** *Microbacterium*, biofilm, psychrotrophic, spoilage, shelf life, micro-filtered milk

## Abstract

The microbiota that spoil long-life micro-filtered milk generally includes species of the genus *Microbacterium*. The metabolic properties of this of microorganisms that could potentially modify the quality of micro-filtered milk are still unexplored when compared to better-known microorganisms, such as the spore-forming *Bacillus* and *Paenibacillus* spp., and Gram-negative contaminants, such as species of the genera *Pseudomonas* and *Acinetobacter*. In this preliminary study, two strains of *Microbacterium* (*M. lacticum* 18H and *Microbacterium* sp. 2C) isolated from micro-filtered milk were characterized in depth, both phenotypically and genotypically, to better understand their role in long-term milk spoilage. The study highlights the ability of these strains to produce high cell numbers and low acidification in micro-filtered milk under storage and shelf-life conditions. Phenotypic analyses of the two *Microbacterium* sp. isolates revealed that both strains have low proteolytic and lipolytic activity. In addition, they have the ability to form biofilms. This study aims to be a preliminary investigation of milk-adapted strains of the *Microbacterium* genus, which are able to grow to high cellular levels and perform slight but not negligible acidification that could pose a potential risk to the final quality of micro-filtered milk. Furthermore, *M. lacticum* 18H and *Microbacterium* sp. 2C were genotypically characterized in relation to the characteristics of interest in the milk environment. Some protein-encoding genes involved in lactose metabolism were found in the genomes, such as β-galactosidase, lactose permease, and L-lactate dehydrogenase. The phenotypically verified proteolytic ability was supported in the genomes by several genes that encode for proteases, peptidases, and peptide transferases.

## Introduction

Micro-filtration and heat treatment are established processes that are often used in combination to increase the shelf life of milk. Specifically, pasteurization treatments or high-temperature pasteurization treatments, in addition to maintaining the cold chain, can extend the shelf life of milk by 1–4 weeks (Schmidt et al., 2012). Usually, the shelf life of drinking milk, called “micro-filtered milk,” ranges from 14 to 21 days after packaging under refrigeration conditions (Caplan and Barbano, 2013). Micro-filtered milk has an extremely low microbiological load. However, at the end of the milk’s shelf life, it cannot be excluded that some microorganisms may have reached high cell numbers. The main spoilage bacterial groups are represented by both Gram-positive and Gram-negative species, especially those of the following genera: *Microbacterium*, *Bacillus* and *Paenibacillus* (the spore-forming species), *Pseudomonas*, *Acinetobacter*, *Chryseobacterium*, *Psychrobacter*, and *Sphingomonas* (Schmidt et al., 2012; Doll et al., 2017). Our previous research on Italian micro-filtered milk, conducted by sampling freshly packaged milk in three different production plants at different times of the year, has shown, through culture-dependent techniques and 16S rRNA gene sequencing of isolates, that the genus *Microbacterium* is dominant among spoilage microorganisms (unpublished data). Previous studies have shown that species of the genus *Microbacterium* exhibit heat resistance that allows them to survive pasteurization treatments (Anagnostopoulos et al., 1964; Washam et al., 1977). In contrast, Gram-negative bacteria are generally cross-contaminants that appear in milk after micro-filtration and pasteurization (Doll et al., 2017). *Microbacterium* spp. are coryneform bacteria that belong to the phylum *Actinobacteria*, the order *Actinomycetales*, and the family *Microbacteriaceae* (Collins-Thompson et al., 1972; Bernard, 2012). Several studies have identified strains belonging to the genus *Microbacterium* in milk after pasteurization and after the milk’s shelf life at refrigeration temperatures. For example, the heat-resistant and psychotropic strains *Microbacterium lacticum* and *Microbacterium flavum* were isolated from pasteurized milk after a prolonged storage period (14 days) at 7.2°C (Washam et al., 1977). Given the high thermal stability of the *M. lacticum* strains isolated from pasteurized milk, they have also been used for thermal resistance experiments, in combination with an extrusion process, in which they were subjected to both mechanical and thermal energy (Bulut et al., 1999). *Microbacterium* spp. are often isolated from the psychrophilic microbiota of raw milk (von Neubeck et al., 2015; Machado et al., 2017; Yuan et al., 2019) and are characterized as highly proteolytic, lipolytic microorganisms (Hantsis-Zacharov and Halpern, 2007; Baur et al., 2015). However, it has been shown that from the time of contamination until the end of the shelf life of micro-filtered milk, *Microbacterium*’s proteolytic and lipolytic activities are below the threshold of detection (Schmidt et al., 2012). Regarding dairy products, it is interesting to note that strains of the genus *Microbacterium* are often found on the rind of smear-ripened cheeses (Mounier et al., 2005, 2007) and that the community of psychrophilic bacteria in raw milk, including *Microbacterium*, has the ability to form biofilms along the whole milk chain (e.g., on the collection and storage equipment), which poses a risk to the stability of the milk (Weber et al., 2019). However, no data are available on the genomic structures underlying the phenotypes of the genus *Microbacterium* that are involved in the preservation of extended shelf-life (ESL) milk.

The aim of this study was to investigate two strains of *Microbacterium* spp. isolated from micro-filtered milk and to use them as strains representing the subpopulation of microorganisms belonging to the genus *Microbacterium*. Their phenotypic and genotypic characteristics were assessed in relation to the milk environment to investigate whether they can be considered a risk to milk quality. In particular, the growth potential and acidification power under low temperature conditions (8°C) were tested by simulating milk shelf life, which can vary between 14 and 21 days (Caplan and Barbano, 2013). Acidification activity, proteolysis, lipolysis, and biofilm formation were evaluated. The study also increased the knowledge of the milk-adapted *Microbacterium* genus, which is still poorly characterized, both phenotypically and genotypically.

## Materials and Methods

### Cultivation of the Strains

The bacteria used in this study were two *Microbacterium* strains, 18H and 2C, isolated from samples of freshly packaged micro-filtered milk, which were chosen as representatives of the two different dominant morphologies. An amount of 100 ml of micro-filtered milk was plated in 10 Petri dishes (150 mm) on milk plate count agar (MPCA) supplemented with triphenyl tetrazolium chloride (TTC) at a final concentration of 0.1 g/L to color the bacterial colonies. The 16S rRNA sequencing analysis was used to confirm that the two strains belonged to the *Microbacterium* genus. During the experimental tests, the strains were grown at temperatures of 30 and 8°C in tryptic soy broth (TSB) and skim milk (SM), respectively. The non-selective medium MPCA was used to measure the growth of the strains, using the tenfold serial dilution technique following incubation at 30°C for 48 h in aerobic conditions. The final value was expressed in colony-forming unit/milliliter (cfu/ml). The type strain *M. lacticum* DSM 20427 was used as a control strain and it was cultured under the same conditions as the tested isolates.

In addition, the proteolytic and lipolytic bacterium *Pseudomonas fluorescens* ATCC 3525 (Oliveira et al., 2018) was used as a positive control during the evaluation of proteolytic and lipolytic activity, while the biofilm producer *Listeria innocua* ATCC 33090 (Perni et al., 2006) was utilized as a positive control in the Congo red assay. *Escherichia coli* ATCC 25922 were used as a reference strain to evaluate the fermentation of lactose, glucose, and galactose. These strains were grown using TSB.

### Taxonomic Identification Through 16S rRNA Gene and Whole-Genome Sequencing

The DNA of the strains under study – *M. lacticum* 18H, *Microbacterium* sp. 2C, and the type strain *M. lacticum* DSM 20427 – was extracted with microLYSIS® (Labogen, London, UK). The 16S rRNA gene sequences were amplified with P0 (5'-GAG AGT TTG ATC CTG GCT-3') and P6 (5'-5'CTA CTA CCT TGT TAC-3') oligonucleotides. The products were purified and sequenced with Sanger technology (Rebecchi et al., 2015). The species were identified by aligning the sequences with the Ribosomal Database Project (RDP) and National Center for Biotechnology Information (NCBI) databases using a naïve Bayesian classifier (Wang et al., 2007). Genomic DNA was also extracted from the same strains for whole-genome sequencing (WGS) with the MasterPure™ Gram-positive DNA Purification Kit (Lucigen Corporation, Middleton, WI, United States), according to the protocol supplied. The quality of the extracted DNA was verified with agarose gel electrophoresis (1%), and the quantity was measured with a Qubit fluorometer (Life Technologies, Carlsbad, CA, United States). Genomic DNA was sequenced on the Illumina NextSeq platform, with the NextSeq High Kit (Illumina Inc., San Diego, CA, United States) to prepare a library (150 + 150 bp). *De novo* genome assemblies were identified with the Shovill pipeline, version 0.9,^1^ and assembled with the SPAdes tool (3.12.0), using the default k-mer sizes 31, 51, 71, and 91 and 111 parameters (Bankevich et al., 2012). The quality of the assembled genomes was evaluated with the QUAST tool (v4.6.0; Gurevich et al., 2013). The species were investigated with the FastANI tool to compute the average nucleotide identity (ANI), with 95% similarity as the threshold.

### Evaluation of Growth in Milk and pH Changes in Milk Under Extended Shelf-Life Conditions

*Microbacterium lacticum* DSM 20427 and the two new strains, 18H and 2C, were assayed for their ability to grow in milk. An overnight culture of each strain was inoculated at 30°C in TSB. After 12 h, the cells (1% v/v) were inoculated into skim milk to adapt to the milk environment and incubated for 24 h at 30°C. These skim milk cultures were used to inoculate other batches of skim milk, obtaining a concentration of about ~10^2^–10^3^ cfu/ml to evaluate growth kinetics and pH. The growth rate was calculated with DMFit ComBase, based on triplicate cultures (Baranyi and Roberts, 1994). Serial dilutions of milk samples were carried out in a sterile peptone solution (1% w/v) and plated on trypticase soy agar (Oxoid, UK). The plates were incubated at 30°C for 48 h, and the resulting colonies were counted. At the same time, the pH was measured with a pH meter (sensION + PH3 basic pH benchtop meter; Crison, Spain).

### Lactose, Galactose, and Glucose Fermentation Assays

The fermentation of sugars was tested with a culture medium consisting of 0.5 g/L of L-cysteine, 20 g/L of tryptone, 5 g/L of NaCl, 0.5 g/L of NaSO_3_, 0.17 g/L of phenol red, and 10 g/L of the sugar to be tested. Then, the culture media containing glucose, galactose, and ribose were autoclaved at 121°C for 15 min. The medium containing lactose, in contrast, was sterilized by filtration using a 0.45-μm filter to avoid sugar degradation. The tests were conducted in duplicates in 96-well plates, with two batches of the same inoculum, as shown in Supplementary Figure 1A. A sugar-free culture medium was used as a negative control, and *E. coli* ATCC 25922 was used as a positive control. To control for the possibility of contamination, some wells were filled with medium only, without inoculation. As *M. lacticum* DSM 20427 does not produce organic acids from ribose (Goodfellow, 2012), the ability to ferment ribose was tested as a negative control. A similar test was carried out with a skim milk culture medium in which 0.17 g/L phenol red was added to confirm the color change to red of yellow in the milk matrix.

### Proteolytic and Lipolytic Activity Assays

Proteolytic activity was measured on skim milk agar, prepared at a concentration of 5% skim milk. The proteolytic activities of the two isolates were evaluated with the spot technique, and the halo of proteolysis was evaluated after 72 h. Lipolytic activity was assessed on tributyrin agar with the spot technique, and the halo of lipolysis was also evaluated after 72 h.

### Biofilm Formation Assay

Biofilm formation was assessed with the Congo red assay (Freeman et al., 1989). We prepared the medium by adding 0.8 g/L Congo red dye to MPCA supplemented with 5% (w/v) sucrose, and the plates were incubated for 48 h at 30°C. The appearance of a black stain was considered a positive result. In addition, we tested the biofilm formation ability on a polystyrene 96-well plate and on sterile stainless-steel spheres (0.5 mm diameter) in TSB and skim milk culture media. Biofilm formation was evaluated after incubation for 72 h at 30°C, after which unattached cells at the bottom of the wells or stainless-steel beads were washed with sterile bi-distilled water. The remaining attached cells were marked with 0.3% crystal violet. We removed the excess crystal violet solution by washing with bi-distilled water; the attached cells were resuspended with a 10% acetic acid solution and quantified by a spectrophotometric measurement at 590 nm, as described by Bassi et al. (2017) with some modifications. To assess whether these strains showed significant differences in their ability to form biofilms (crystal violet staining method), 12 inoculation replicates were made, and an ANOVA was carried out with Tukey’s honestly significant difference test.

### Cell Size Measurement and Biofilm Matrix Visualization Through Scanning Electron Microscopy Analysis

Samples were prepared for scanning electron microscopy (SEM) as follows: 5 ml of microbial cells was grown in TSB at 30°C, up to the stationary phase. Then, 5 μl of the cultures was fixed on a positively charged nylon membrane (Roche Diagnostics GmbH, Germany) and treated as described by Bassi et al. (2009). The image resulting from the SEM analysis was processed with ImageJ (Rueden et al., 2017) to measure the length and width of the cells. In addition, the image was evaluated visually to confirm the presence of any extracellular polymeric matrices associated with biofilm formation.

### Investigation of Genomic Features by Bioinformatic Analyses

The assembled genomes used to investigate the species were annotated with Prokka (v1.13.3; Seemann, 2014). The presence of complete prophage regions in the genomic sequences was evaluated with the PHASTER tool. The mobile elements and CRISPR and Cas genes were detected with ISFinder and CRISPRCasFinder, respectively. Genes potentially involved in antibiotic resistance were scanned through the Resistance Gene Identifier (RGI) database. The assembled genome of *M. lacticum* DSM 20427 (accession number ASM671681v1) was processed, as described above, for the comparison of the genotypic properties of our strains. The genome sequences of the strains used in this study were deposited in the NCBI repository, with the accession numbers listed in Table 1.

**Table 1:** General features of the three genomes of *Microbacterium*.

Strain	Accession number NCBI	The number of contigs	N_50_	Estimated genome size (bp)	GC content (%)	CDS	tRNA coding genes	rRNA coding genes
*Microbacterium lacticum* DSM 20427	ASM671681v1	9	613,777	3,086,900	70.30	2,980	47	3
*Microbacterium lacticum* 18H	JAAGRZ000000000	367	27,772	3,302,182	69.69	3,199	52	4
*Microbacterium* sp. 2C	JAAGRY000000000	142	40,778	3,215,958	69.76	3,049	55	2

## Results and Discussion

### Taxonomic Identification

Identification by amplification of the 16S rRNA gene using the P0 and P6 oligonucleotides produced a trimmed amplicon of about 800 bp. It is important to note that previous studies (Takeuchi and Hatano, 1998) have confirmed the difficulty in identifying species of the genus *Microbacterium* based on the 16S rRNA gene sequence. For this reason, we carried out WGS on our isolates. Species identification was performed according to the workflow proposed by Chun et al. (2018). Specifically, a complete 16S rRNA gene sequence was used and recovered from WGS to perform alignments with longer sequences (i.e., over 1,500 bp) in the database. The resulting blast shows many species of *Microbacterium* with >98.7% similarity. The resulting 16S rRNA gene identity percentages with respect to the 18H strain were as follows: *M. lacticum* 99.80%, *Microbacterium schleiferi* 99.11%, *Microbacterium saccharophilum* 98.72%, *M. flavum* 98.70%, and *Microbacterium aoyamense* 98.69%. The resulting 16S rRNA gene identity percentages with respect to the 2C strain were as follows: *M. lacticum* 99.25%, *M. flavum* 99.24%, *M. schleiferi* 99.04%, *M. aoyamense* 99.03%, *M. saccharophilum* 98.92%, *Microbacterium lacus* 98.91%, *Microbacterium pumilum* 98.86%, *Microbacterium diaminobutyricum* 98.80%, *Mycobacterium aurum* 98.71%, and *Microbacterium deminutum* 98.68% (Supplementary Table 1). We calculated the ANI to achieve a high-resolution identification and identified the 18H strain as *M. lacticum*, with 96% sequence similarity to *M. lacticum* DSM 20427. In contrast, none of the reference genomes used to calculate the ANI provided a species-level identification of the 2C strain (ANI values <95%; Supplementary Table 2).

### Ability to Grow in Milk and Acidification Activity

**Figure 1:**
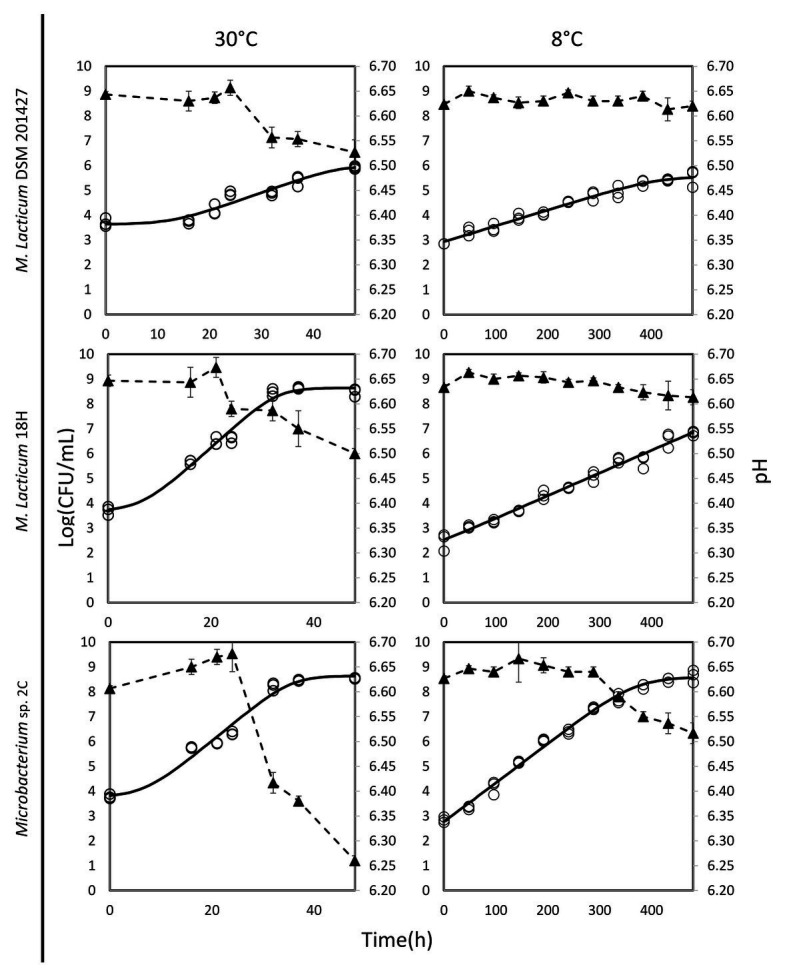
Dynamics of growth and acidification in milk of *M. lacticum* DSM 20427, *M. lacticum* 18H, *Microbacterium* sp. 2C at two different temperatures, 30 and 8°C. The growth results were based on triplicate measures (○). The continuous line (-) shows the growth curve resulting from the DMFIT (Baranyi and Roberts, 1994). The acidification curve (▲, ‐ -) is expressed as mean values from triplicate measures.

The ability of *Microbacterium* isolates to grow in milk was assessed at 30 and 8°C. At 30°C, the *M. lacticum* 18H strain exhibited the fastest growth rate in milk. Similar growth performance at 30°C in milk was also shown by the *Microbacterium* sp. 2C strain, which was slightly slower, while the type strain, *M. lacticum* DSM 20427, used as a comparison strain, showed a slower growth rate and reached a final cell count that was 2 logs lower than the cell counts of the two strains isolated from micro-filtered milk (Figure 1; Table 2). At 8°C, the growth rate was generally reduced for all strains compared to the test conducted at 30°C. The *M. lacticum* DSM 20427 strain confirmed the weak ability of growth to grow in milk even at low temperatures. On the other hand, the strains isolated in this study, *M. lacticum* 18H and *Microbacterium* sp. 2C, showed an inverse trend compared to the test at 30°C. Under these experimental conditions, *M. lacticum* 18H demonstrated a lower growth capacity and growth rate compared to *Microbacterium* sp. 2C. The latter strain was able to reach the highest cfu levels, which were about 2 logs higher than those of *M. lacticum* 18H and about 3 logs higher than those of *M. lacticum* DSM 201427 (Figure 1; Table 2).

**Table 2:** Summary of the recorded data obtained by Combase: the maximum growth rate (Log cfu/ml/h), the final value (Log cfu/ml), and the goodness of the fit data (R-square).

Combase output	*M. lacticum* DSM 20427		*M. lacticum* 18H		*Microbacterium* sp. 2C	
	30°C	8°C	30°C	8°C	30°C	8°C
R-square	0.90	0.96	0.97	0.97	0.97	0.99
Maximum rate	0.0743 ± 0.0100	0.00638 ± 0.0003	0.187 ± 0.0180	0.0092 ± 0.0006	0.1690 ± 0.0160	0.0159 ± 0.0006
Final value	5.99 ± 0.22	5.57 ± 0.10	8.64 ± 0.12	6.99 ± 0.38	8.64 ± 0.15	8.59 ± 0.08

The evaluation of the pH of milk in the presence of the three strains studied after a 48-h incubation period at 30°C showed a decrease of about 0.1 pH units, a decrease of about 0.15 pH units for the strains *M. lacticum* DSM 20427 and 18H, and a decrease of about 0.4 pH units for the strain *Microbacterium* sp. 2C. During incubation at 8°C, the pH of milk incubated with *M. lacticum* DSM 20427 and *M. lacticum* 18H remained unchanged after 480 h (20 days). In contrast, the strain *Microbacterium* sp. 2C produced slight acidification of the milk, which lowered the pH by about 0.1 units in 20 days (Figure 1). Our data indicated that *Microbacterium* grew efficiently in milk at 30°C. In addition, both strains of *Microbacterium* grew to high cfu levels when incubated at 8°C for 20 days. These results clearly show that the studied strains isolated from micro-filtered milk are able to grow in milk under low temperature conditions and that they can be considered psychrotrophic microorganisms. Their ability to grow under suboptimal conditions suggests that they could pose a serious risk to the stability of micro-filtered milk during its shelf life – the total bacterial count at the end of the simulated shelf life reached high values, and the pH also underwent slight, but relevant, decreases.

### Lactose, Galactose, and Glucose Fermentation

When studying the adaptation of microorganisms to the milk environment, it is essential to evaluate their ability to break down lactose and to use the resulting glucose and galactose for metabolic activity. The lactose fermentation test showed that *M. lacticum* DSM 20427 and *Microbacterium* sp. 2C were able to break down lactose into glucose and galactose, but *M. lacticum* 18H was found to be a weak lactose fermenter. The ability to use lactose was confirmed by investigating which of the two monomers were best fermented by the microorganisms under study. The galactose fermentation test showed that *M. lacticum* DSM 20427 and *M. lacticum* 18H were weak fermenters of galactose, while *Microbacterium* sp. 2C failed to ferment galactose (negative results of the fermentation test). Glucose was fermented by all strains tested (Supplementary Figure 1A). The same test was conducted in the milk environment. All three inoculated strains showed positive test results, as shown in Supplementary Figure 1B, which confirmed the results of previous tests conducted with individual sugars. *Microbacterium*’s ability to use lactose was consistent with the microbial cell growth values measured in milk incubated at 30 and 8°C. However, the use of lactose was not efficient in terms of conversion into acidification-inducing organic acids typical of fermentative metabolism. In fact, the measured pH values showed a weak acidification activity for *Microbacterium* sp. 2C and no acidification activity for *M. lacticum* 18H and DSM 20427 (Table 3).

**Table 3:** Summary of the phenotypic characterization of the *Microbacterium* strains.

Strain	Proteolytic activity assay	Lipolytic activity assay	Congo red method	Glucose fermentation	Galactose fermentation	Lactose fermentation
	Clear zone	Clear zone	Black colony			
*M. lacticum* DSM 20427	+	+	+	+	+/−	+
*M. lacticum* 18H	+	+	+	+	+/−	+/−
*Microbacterium* sp. 2C	+	−	+	+	−	+

Regarding the proteolysis and lipolysis tests, the symbols “+” and “−” represent, respectively, the presence or absence of a transparent halo indicating enzymatic activity, while for the Congo red test, the presence of black colonies indicates the potential to produce biofilms. Regarding the fermentation of sugars, the symbols “+,” “+/−,” and “−” represent the change of color, as measured by the pH indicator (phenol red) inside the culture medium, from red (no acidification) to orange (low acidification) to yellow (acidification).

### Proteolytic and Lipolytic Activities

*Microbacterium* is a contaminant of micro-filtered milk (Schmidt et al., 2012). Therefore, its potential proteolytic and lipolytic activities are of considerable importance to the final milk quality. Because of the action of proteases and lipases, milk can undergo changes that alter its quality, with the result that it cannot reach the end of its shelf life in good commercial conditions. A previous study carried out on the psychrotrophic microbiota in raw milk showed that the majority of isolated *Microbacterium* species had both proteolytic and lipolytic activities (Vithanage et al., 2016). In our study, none of the strains investigated showed strong proteolytic activity, particularly compared to known proteolytic microorganisms such as *P. fluorescens*, which was used as a positive control in our experiments. *M. lacticum* DSM 20427 and *Microbacterium* sp. 2C showed qualitatively slight proteolysis, but the *M. lacticum* 18H strain displayed a weaker proteolysis activity than the other two strains (Table 3; Supplementary Figures 2A,B). Similarly, the strains tested had a slight lipolytic activity not comparable to that of the reference strain (i.e., *P. fluorescens*). Nevertheless, both *Microbacterium* sp. 2C and *M. lacticum* 18H displayed the ability to grow in milk at 8°C (Figure 1). This result suggests that even weak proteolytic and lipolytic activity could seriously threaten the stability and quality of micro-filtered milk at the end of its shelf life.

### Biofilm Formation

**Figure 2:**
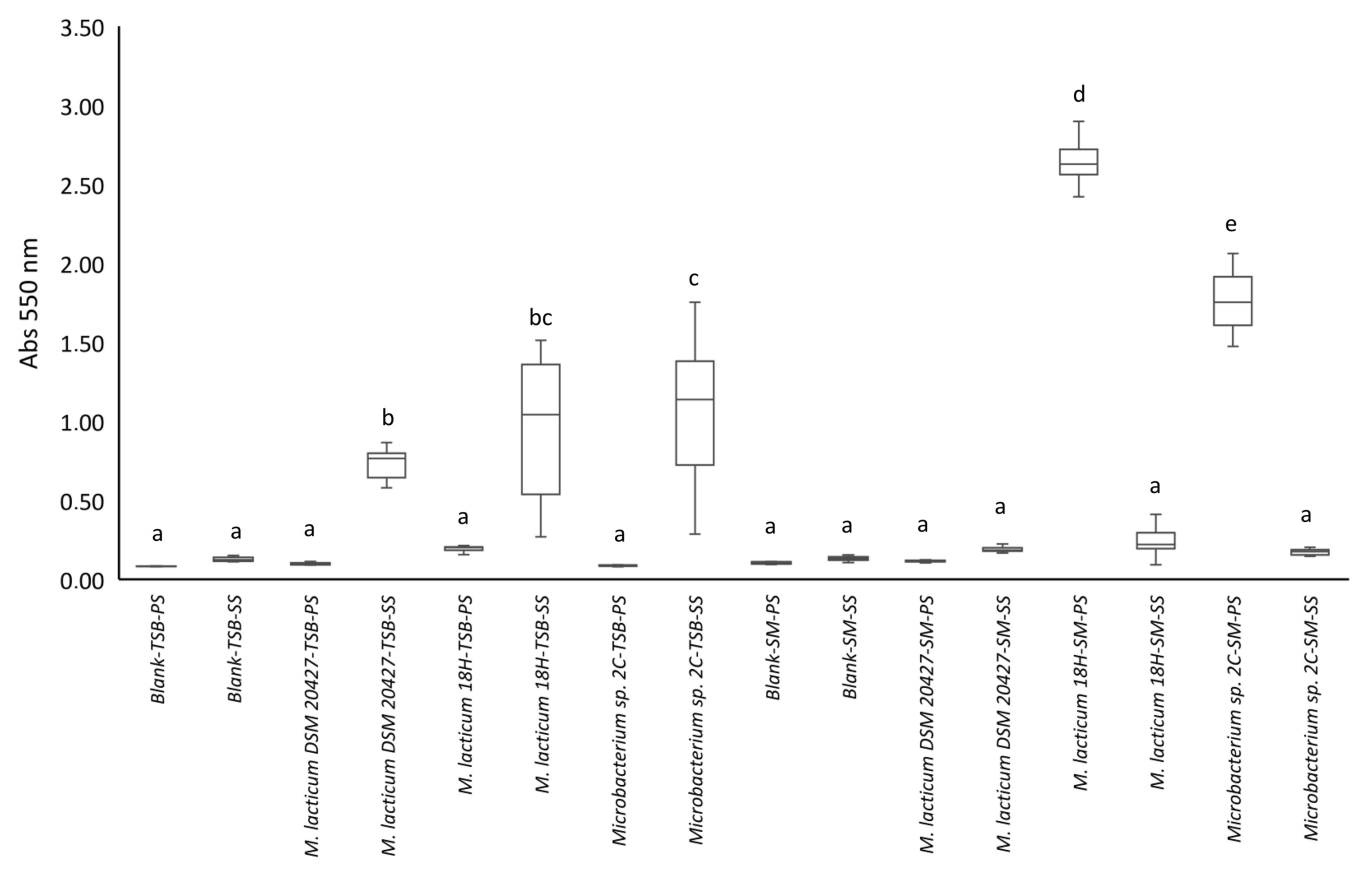
Crystal violet staining quantification by measuring absorbance (Abs_550 nm_). Values <0.1 represent no biofilm formation; values between 0.1 and 0.25 indicate a low biofilm formation; values between 0.25 and 0.6 indicate a medium biofilm formation, whereas values >0.6 represent a high biofilm formation. PS, polystyrene; SS, stainless steel; TSB, tryptic soy broth; SM, skim milk.

**Figure 3:**
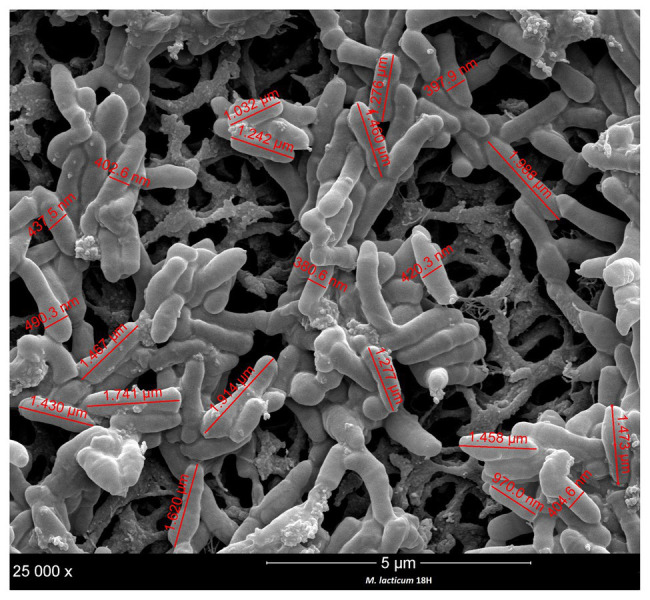
Size and cell measurement of *M. lacticum* 18H by scanning electron microscopy (SEM) observation.

**Figure 4:**
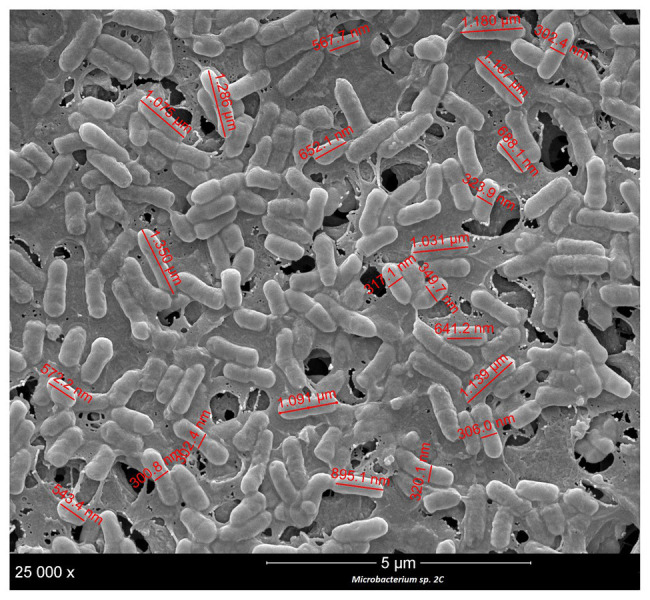
Size and cell measurement of *Microbacterium* sp. 2C by SEM observation.

We evaluated the biofilm formation ability because this process could explain why *Microbacterium* is a frequent contaminant of micro-filtered milk. In particular, the ability to adhere to the steel used in micro-filtered milk production plants and milk collection tanks could favor the spread of this bacterium. The appearance of black staining around the colonies grown in the Congo red medium was considered a positive indication of the ability to form biofilms (Supplementary Figure 2C). We found that all strains showed the change from red to black. The ability of bacterial cells to adhere and potentially form biofilms was tested in 96-well plates, with and without stainless steel spheres, with TSB as the culture medium. All tested strains showed a significant difference in adhesion to steel spheres compared to adhesion to the polystyrene well surface (Figure 2). All three strains formed biofilms on steel, but *Microbacterium* sp. 2C formed significantly more biofilms than *M. lacticum* 18H and *M. lacticum* DSM 20427. In contrast, when skim milk was used as the culture medium, none of the strains adhered to steel spheres, but *M. lacticum* 18H and *Microbacterium* sp. 2C showed significant adherence to polystyrene (Figure 2). Therefore, our results show that *Microbacterium* cannot form biofilms on steel in a milk environment. However, in a more nutritive environment, such as the TSB medium, *Microbacterium* displayed an excellent biofilm-forming ability. Our SEM images showed that *M. lacticum* 18H cells, grown in the TSB medium, showed only a weak production of extracellular material (Figure 3). In contrast, *Microbacterium* sp. 2C cells formed a biofilm visible during the stationary phase (Figure 4), i.e., we could observe a polymer matrix covering the cells.

### Cell Size Measurement

In the processing of ESL milk, micro-filtration could be a critical step in the sanitization of raw milk. Accordingly, we decided to evaluate the cell sizes of the two strains under study, *M. lacticum* 18H and *Microbacterium* sp. 2C. We measured cell sizes by examining SEM images, and both strains showed short, polymorphic bacilli morphologies, which were slightly covered with a polymeric matrix. The cells of *M. lacticum* 18H had a mean length of 1.340 ± 0.5 μm and a mean diameter of 0.410 ± 0.4 μm; the *Microbacterium* sp. 2C cells had a mean length of 0.890 ± 0.5 μm and a mean diameter of 0.320 ± 0.3 μm. The ceramic membranes of micro-filtration plants generally have pore diameters of 1.4 μm (Fernández García et al., 2013); therefore, our measurements of *M. lacticum* 18H and *Microbacterium* sp. 2C cells indicate that these cells could pass through the micro-filtration membranes (Figures 3, 4).

### Genome Analysis

#### General Features

The general characteristics of the three sequenced genomes are shown in Table 1. All *Microbacterium* strains were comparable in genome size and guanine-cytosine (GC) content (~70%). Plasmids were absent in all genomes, and complete prophage regions were identified only in *M. lacticum* DSM 20427. Sequences related to CRISPR were also found: three in *M. lacticum* DSM 204272, two in *M. lacticum* 18H, and four in *Microbacterium* sp. 2C. In contrast, there were no Cas-protein-encoding regions. Regarding mobile elements, 87 transposons that belonged to 9 insertion sequence (IS) families (ISL3, IS256, IS256, IS3, IS481, IS110, IS30, Tn3, and IS1595) were present in *M. lacticum* DSM 20427; 14 transposons that belonged to one IS family (IS481) were identified in *Microbacterium* sp. 2C; and only one transposon was found in *M. lacticum* 18 h. Moreover, no antibiotic resistance genes were detected in any of the three genomes.

#### Lactose-Utilization Features

To support the phenotypic evidence that these bacteria utilized lactose, the genes that resulted from the Prokka annotation (Supplementary Table 3) were investigated for links with lactose and galactose metabolism. Our Prokka annotation revealed that all the *Microbacterium* strains investigated could hydrolyze lactose to glucose and galactose through the enzyme β-galactosidase. Interestingly, only the 2C and 18H strains had genes that encode proteins for the lactose internalization system, namely *lacS*, *lacF*, and *lacG*. In addition, the 2C strain had four copies of the *lacF* gene. All strains had a putative gene that encodes the lactose operon repressor, which is activated in the presence of lactose. Furthermore, galactose-1-phosphate uridylyltransferase, which catalyzes the conversion of galactose to glucose, was present in all three strains. To explain the strains’ acidification ability, we confirmed that all strains had *ldh* and *ldd* genes, which encode L-lactate dehydrogenase. This enzyme converts pyruvate to L-lactate. All strains also carried the *ldr* gene, which regulates the activity of this enzyme.

#### Proteolytic and Lipolytic Features

The potential proteolytic and lipolytic activities of a microorganism are of considerable importance in the milk environment. All genomes of the three *Microbacterium* strains investigated carry genes that encode ATP-dependent proteases, namely *clpS*, *clpC1*, *clpX*, *clpP1*, *clpP2*, and *ftsH*. In addition, all genomes of *Microbacterium* strains under study carry the rhomboid protease (GluP) and the two zinc metalloproteases. However, in this study, only *Microbacterium* sp. 2C carried a putative cysteine protease (YraA). In contrast, all three strains carried both genes that encode members of the peptidase system – including *pepA*, *pepN*, *pepO*, and *pepPI* – and the *prlC* oligopeptidase. Peptide transport mechanisms were represented by genes that encode members of the *Opp* system, including *oopD*, *oppF*, and *oppA*. In addition, we found the gene that encodes the di/tripeptide transporter (*dtpT*). All three strains possessed the gene that encodes monoacylglycerol lipase, and *Microbacterium* sp. 2C also carried a lipase gene (*lip3*; Supplementary Tables 4, 5).

**Table 4:** General features of the three genomes of *Microbacterium*.

Isolate/sequence reference	Prophage region	CRISP/Cas	Mobile elements
*Microbacterium lacticum* 18H	1	2/0	14
*Microbacterium lacticum* DSM 20427	0	3/0	87
*Microbacterium* sp. 2C	0	4/0	1

#### Adhesion and Biofilm Features

Phenotypic tests on the three strains showed different approaches to forming biofilms, which is an important characteristic of spoilage microorganisms found in milk plants. Our genomic analyses identified a few genes that could be related to the ability to form biofilms. All three *Microbacterium* strains carried the *lptB* (lipopolysaccharide export system ATP-binding protein) and *glf* (UDP-galactopyranose mutase) genes, which could be involved in biofilm formation. *M. lacticum* DSM 204272 also carried the *epsF* gene (type II secretion system protein F), while *Microbacterium* sp. 2C carried the *epsL* gene, which encodes a sugar transferase. The *epsF* gene encodes glycosyltransferase, thus contributing to exopolysaccharide biosynthesis (Nielsen et al., 2019). The role of *epsL* gene in the formation of biofilms and the involvement of the *eps* gene in surface colonization were shown in *Bacillus subtilis* (Nagórska et al., 2010; Supplementary Table 5).

#### Temperature-Related Features

*Microbacterium* is considered a heat-resistant microorganism (Bulut et al., 1999; Walsh et al., 2012). Its genome has a high GC content. We measured GC values of 69.69 and 70.30 for *M. lacticum* 18H and *Microbacterium* sp. 2C, respectively, which confirmed their heat resistance. From the annotations, in all three strains we found genes that encode heat shock proteins, including chaperonins – *hslR*, *grpE*, *dnaK*, and *dnaJ* – in addition to the chaperone gene *clpB*, which encodes a caseinolytic protease. In addition, we identified genes putatively involved in the response to both thermal and cold stress (*clpX* and *clpS*). In terms of psychrotrophic features, all three strains carried the *cspA* gene (cold shock protein; Supplementary Table 5).

## Conclusion

The technology used to prepare micro-filtered milk exerts strong selection pressures on the spoilage microbiota of raw milk. For survival, these strains must pass through micro-filtration and tolerate thermal stress at high temperatures. In this study, thanks to the use of two representative strains isolated from micro-filtered milk and belonging to the *Microbacterium* genus, it was investigated the spoilage-potential of two species isolated from the milk environment. The genome analyses showed that the two strains carried genes that conferred tolerance to the thermal stresses they were exposed to, during milk production. Moreover, SEM analysis showed that both strains were small enough to cross the micro-filtration barrier. The genetic analyses and phenotypic tests showed that the strains could utilize lactose for energy. They also had the capacity to degrade proteins and lipid components. Moreover, we found that they could grow in milk under low temperatures. Finally, we demonstrated that these strains could form biofilms, but this ability was not confirmed for steel surfaces. Based on these findings, we conclude that *M. lacticum* 18H and *Microbacterium* sp. 2C can potentially affect the shelf life of micro-filtered milk, as they possess all the characteristics needed to survive the barriers present in the production process. Accordingly, these bacteria may present a risk to the stability of micro-filtered milk by reducing its shelf life. This preliminary work allowed us to understand the behavior of these microorganisms in the milk environment. However, further tests on a higher number of isolates will be necessary to assess the impact of *Microbacterium* spp. on the shelf life of micro-filtered milk. Future studies may investigate changes directly in the milk matrix by measuring the degradation products (such as acidity, free amino acids, and free fatty acids) resulting from the microorganisms’ metabolism to confirm whether the impact on the final product could be considered dangerous or negligible to the quality of micro-filtered milk.

## Data Availability Statement

The datasets presented in this study can be found in online repositories. The names of the repository/repositories and accession number(s) can be found in the article/Supplementary Material.

## Author Contributions

PB carried out the cultural, molecular, and bioinformatics analyses and wrote the manuscript. AF supervised and performed the bioinformatics analysis and reviewed the manuscript. FC codesigned the study and reviewed the manuscript. LM codesigned the study and reviewed the manuscript. All authors contributed to the article and approved the submitted version.

### Conflict of Interest

The authors declare that the research was conducted in the absence of any commercial or financial relationships that could be construed as a potential conflict of interest.

## Footnotes

^1^https://github.com/tseemann/shovill
