# Supplementary material for: Phenotypic and Genotypic Investigation of Two Representative Strains of Microbacterium Species Isolated From Micro-Filtered Milk: Growth Capacity and Spoilage-Potential Assessment
Source: Front Microbiol. 2020 Oct 22;11:554178. doi: 10.3389/fmicb.2020.554178 (PMC7642513; doi:10.3389/fmicb.2020.554178)
Supplement: Supplementary file 1 [file Data_Sheet_1.docx]

**Supplementary information**

**Supplementary Figure 1.** Sugar fermentation assay

**Supplementary Figure 2.** Proteolytic, lipolytic and Congo Red agar plate assay

**Supplementary Table 1.** Identifications of 18H and 2C strains resulting from blast alignment of the complete 16s using NCBI 16s type material database Supplementary

**Supplementary Table 2.** ANI values resulting from the comparison of 2C and 18H strains against type material genomes obtained from the NCBI genome database.

**Supplementary Table 3.** Genomic features related to lactose metabolism in *Microbacterium* strains

**Supplementary Table 4.** Genomic features related to protein degradation in *Microbacterium* strains

**Supplementary Table 5.** Other features (lipase, biofilm, heat and cold response features in *Microbacterium* strains

# Supplementary Figures

A


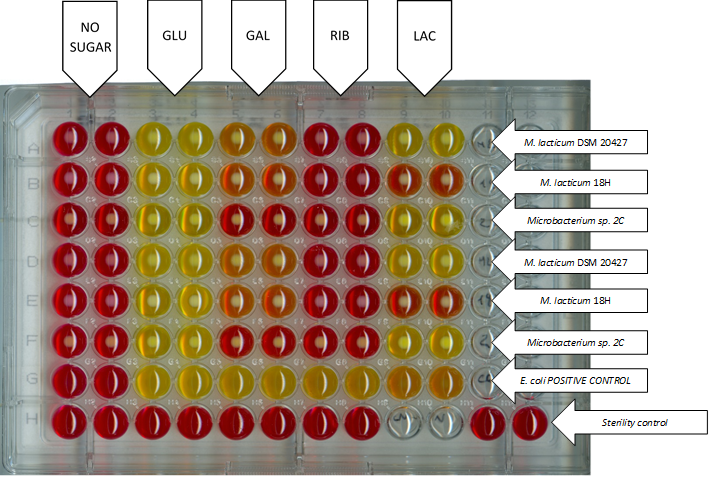


B


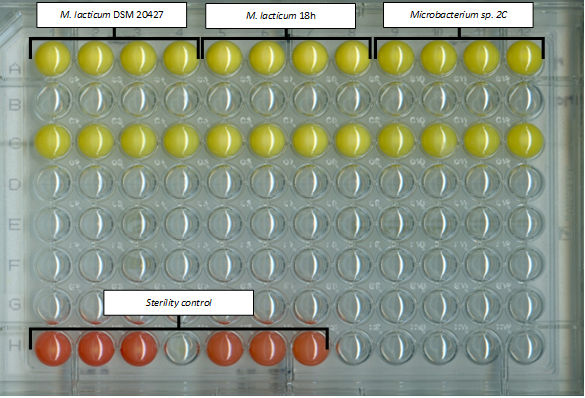
**Supplementary Figure 1.** Sugar fermentation. The yellow colour of the medium represents the positivity of the sugar fermentation. The red colour of the medium shows a negative fermentation. The test was conducted in four replicates of inoculum. In part A of the figure three strains were tested on the single sugars. In part B of the figure the three strains were tested in sterile milk. Phenol red was used as an indicator.


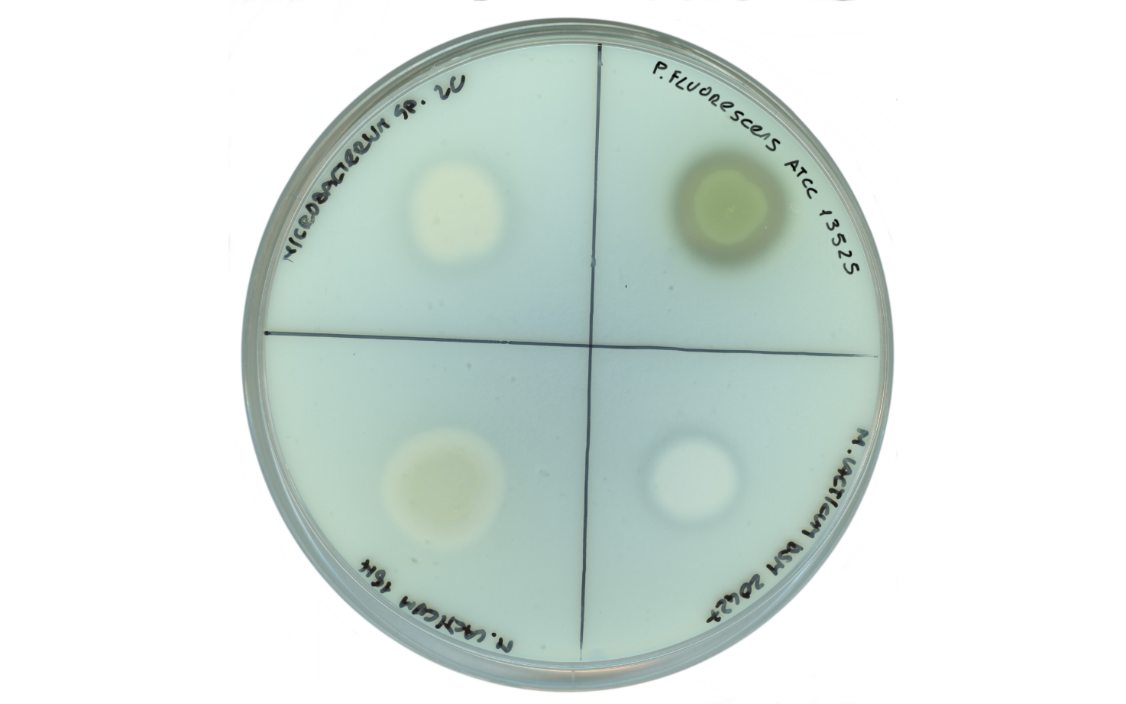

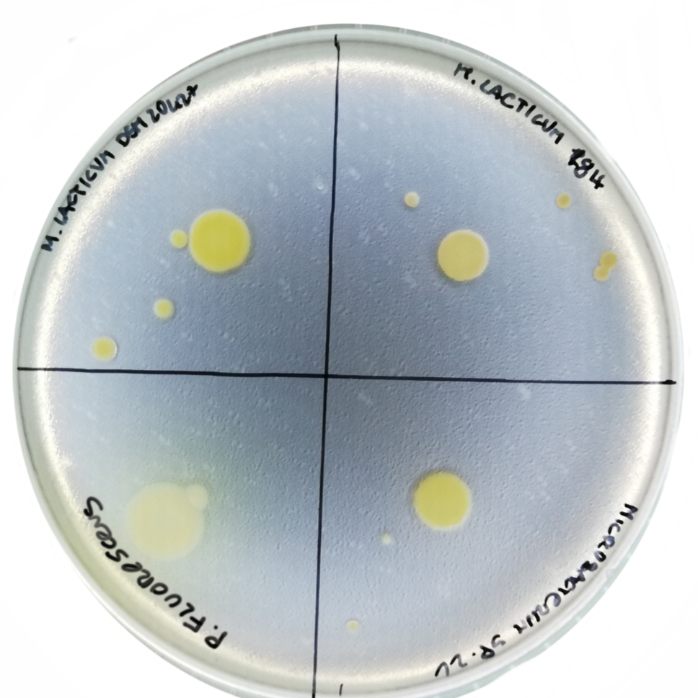


C

B

A


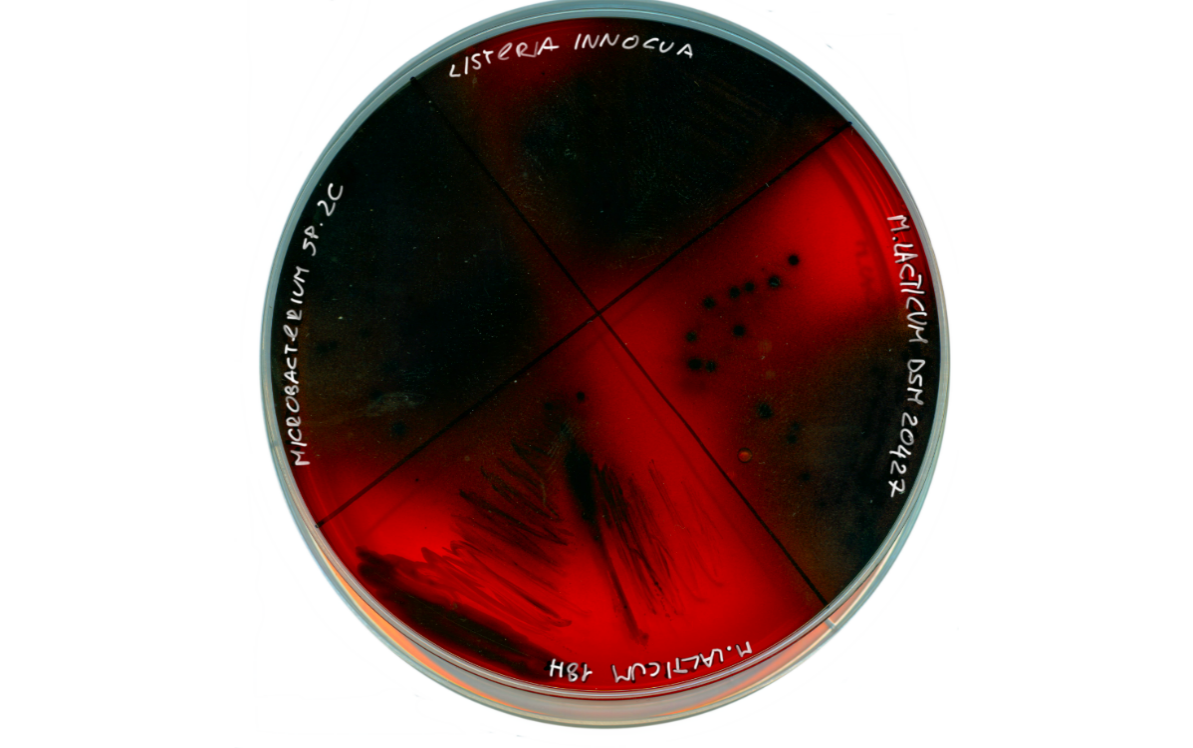


**Supplementary Figure 2.** Proteolytic (A) and lipolytic (B) agar plate assay. The lighter area around the bacterial spot indicates the presence of enzymatic activity. Congo Red (C) agar plate assay. The black colour of the colonies indicates a slime production due to the ability to form biofilms.

# Supplementary Table

**Supplementary Table 1.** Output with an identity >98.6% resulting from the Blast allignement operation against type material of the genus *Microbacterium* NCBI 16S rRNA online database.

| Strain 18H | | | | | | |
| --- | --- | --- | --- | --- | --- | --- |
| Description | Max Score | Total Score | Query | E value | Ident | Accession |
| *Microbacterium lacticum* strain DSM 20427 | 2708 | 2708 | 96% | 0 | 99.80% | NR_026160.1 |
| *Microbacterium schleiferi* strain DSM 20489 | 2627 | 2627 | 95% | 0 | 99.11% | NR_044936.1 |
| *Microbacterium saccharophilum* strain K-1 | 2634 | 2634 | 96% | 0 | 98.72% | NR_114342.1 |
| *Microbacterium flavum* strain YM18-098 | 2582 | 2582 | 94% | 0 | 98.70% | NR_041562.1 |
| *Microbacterium aoyamense* strain KV-492 | 2562 | 2562 | 94% | 0 | 98.69% | NR_041332.1 |
| Strain 2C | | | | | | |
| Description | Max Score | Total Score | Query | E value | Ident | Accession |
| *Microbacterium lacticum* strain DSM 20427 | 2663 | 2663 | 96% | 0 | 99.25% | NR_026160.1 |
| *Microbacterium flavum* strain YM18-098 | 2627 | 2627 | 94% | 0 | 99.24% | NR_041562.1 |
| *Microbacterium schleiferi* strain DSM 20489 | 2621 | 2621 | 95% | 0 | 99.04% | NR_044936.1 |
| *Microbacterium aoyamense* strain KV-492 | 2590 | 2590 | 94% | 0 | 99.03% | NR_041332.1 |
| *Microbacterium saccharophilum* strain K-1 | 2651 | 2651 | 96% | 0 | 98.92% | NR_114342.1 |
| *Microbacterium lacus* strain A5E-52 | 2615 | 2615 | 95% | 0 | 98.91% | NR_041563.1 |
| *Microbacterium pumilum* strain KV-488 | 2505 | 2505 | 91% | 0 | 98.86% | NR_041331.1 |
| *Microbacterium diaminobutyricum* strain RZ63 | 2521 | 2521 | 92% | 0 | 98.80% | NR_152648.1 |
| *Microbacterium aurum* strain DSM 8600 | 2614 | 2614 | 96% | 0 | 98.71% | NR_044933.1 |
| *Microbacterium deminutum* strain KV-483 | 2543 | 2543 | 93% | 0 | 98.68% | NR_041330.1 |

**Supplementary Table 2.** ANI values resulting from the comparison of *Microbacterium* strains 2C and 18H against type material genomes obtained from the NCBI genome database

| Type reference genome material | *Strain* 18H | *Strain*  2C |
| --- | --- | --- |
| *M. lacticum* 18H | 100 | 88.9382 |
| *Microbacterium lacticum* NBRC 14135 | 97.2457 | 88.6876 |
| *Microbacterium lacticum* strain DSM 20427 | 96.9314 | 88.3921 |
| *Microbacterium* sp. 2C | 88.8867 | 100 |
| *Microbacterium aurum* strain KACC 15219 | 84.3124 | 83.7521 |
| *Microbacterium hominis* NBRC 15708 | 83.1925 | 82.9591 |
| *Microbacterium telephonicum* strain S2T63 | 82.7718 | 82.4854 |
| *Microbacterium saccharophilum* NBRC 108778 | 82.3102 | 81.6895 |
| *Microbacterium saccharophilum* strain K-1 | 82.2703 | 81.6881 |
| *Microbacterium xylanilyticum* JCM 13591 | 82.1798 | 81.2226 |
| *Microbacterium ketosireducens* strain DSM 12510 | 81.3091 | 81.4777 |
| *Microbacterium arborescens* strain DSM 20754 | 81.2162 | 80.9675 |
| *Microbacterium paludicola* strain CC3 | 81.1619 | 81.0325 |
| *Microbacterium trichothecenolyticum* strain DSM 8608 | 80.9658 | 80.798 |
| *Microbacterium lemovicicum* strain Viu22 | 80.8606 | 80.612 |
| *Microbacterium oleivorans* NBRC 103075 | 80.7753 | 80.5268 |
| *Microbacterium enclense* strain NIO-1002 | 80.7739 | 80.7615 |
| *Microbacterium ginsengisoli* strain DSM 18659 | 80.757 | 80.8394 |
| *Microbacterium yannicii* PS01 | 80.6991 | 80.5887 |
| *Microbacterium testaceum* StLB037 | 80.6347 | 80.5838 |
| *Microbacterium hatanonis* strain JCM14558 | 80.5139 | 80.3673 |
| *Microbacterium luticocti* DSM 19459 | 80.4762 | 80.4099 |
| *Microbacterium sediminis* strain YLB-01 | 80.3587 | 80.0505 |
| *Microbacterium testaceum* NBRC 12675 | 80.2897 | 80.3884 |
| *Microbacterium chocolatum* strain SIT 101 | 80.2329 | 80.0592 |
| *Microbacterium aerolatum* NBRC 103071 | 80.1695 | 79.7145 |
| *Microbacterium mangrovi* strain MUSC 115 | 80.127 | 80.1045 |
| *Microbacterium pygmaeum* strain DSM 23142 | 80.072 | 80.1375 |
| *Microbacterium azadirachtae* strain DSM 23848 | 80.0106 | 79.9533 |
| *Microbacterium azadirachtae* strain ARN176 | 79.9637 | 79.8335 |
| *Microbacterium hydrocarbonoxydans* strain SA35 | 79.9424 | 80.0203 |
| *Microbacterium hydrocarbonoxydans* NBRC 103074 | 79.9382 | 79.9021 |
| *Microbacterium paraoxydans* NBRC 103076 | 79.9248 | 79.9986 |
| *Microbacterium barkeri* 2011-R4 | 79.9046 | 79.9464 |
| *Microbacterium liquefaciens* NBRC 15037 | 79.8766 | 80.3216 |
| *Microbacterium hydrocarbonoxydans* strain DSM 16089 | 79.8647 | 79.8911 |
| *Microbacterium oxydans* NBRC 15586 | 79.8583 | 79.6034 |
| *Microbacterium oxydans* strain BEL4b | 79.8506 | 79.5073 |
| *Microbacterium resistens* NBRC 103078 | 79.8202 | 79.5964 |
| *Microbacterium profundi* strain Shh49 | 79.7986 | 79.6541 |
| *Microbacterium paraoxydans* strain DSM 15019 | 79.7494 | 79.9176 |
| *Microbacterium maritypicum* MF109 | 79.6982 | 79.4012 |
| *Microbacterium foliorum* strain DSM 12966 | 79.6847 | 79.6915 |
| *Microbacterium foliorum* strain NRRL B-24224 | 79.6832 | 79.6979 |
| *Microbacterium indicum* DSM 19969 | 79.5334 | 79.6788 |
| *Microbacterium gubbeenense* DSM 15944 | 79.5259 | 79.3967 |
| *Microbacterium saperdae* strain DSM 20169 | 79.4761 | 79.725 |
| *Microbacterium esteraromaticum* strain B Mb 05.01 | 79.4597 | 79.4307 |
| *Microbacterium sorbitolivorans* strain C1-15228 | 79.117 | 78.9897 |
| *Microbacterium halotolerans* strain YIM 70130 | 78.924 | 78.9932 |
| *Microbacterium humi* strain DSM 21799 | 78.4662 | 78.5848 |
| *Microbacterium mitrae* strain M4-8 | 78.1127 | 78.338 |
| *Microbacterium lindanitolerans* strain DSM 22422 | 77.398 | 77.8209 |
| *Microbacterium agarici* strain DSM 21798 | 77.3028 | 77.7279 |

**Supplementary Table 3.** Genomic features related to lactose metabolism in *Microbacterium* strains

| Lactose utilization features | | | | | |
| --- | --- | --- | --- | --- | --- |
| Gene | Encoded protein | *M. lacticum* DSM 20427 | | *Microbacterium* sp. 2C | *M. lacticum* 18H |
| *bga* | Beta-galactosidase | | 1 | 0 | 0 |
| *lacZ* | Beta-galactosidase | | 0 | 1 | 0 |
| *cbgA* | Beta-galactosidase | | 0 | 1 | 0 |
| *ebgA* | Evolved beta-galactosidase subunit alpha | | 1 | 0 | 1 |
| *galT* | Galactose-1-phosphate uridylyltransferase | | 1 | 1 | 1 |
| *lacI* | Lactose operon repressor | | 2 | 3 | 2 |
| *lacS* | Lactose permease | | 0 | 0 | 1 |
| *lacF* | Lactose transport system permease protein LacF | | 0 | 4 | 1 |
| *lacG* | Lactose transport system permease protein LacG | | 0 | 1 | 1 |
| *lacE* | Lactose-binding protein | | 0 | 1 | 0 |
| *galK* | Galactokinase | | 2 | 1 | 1 |
| *ldh2* | L-lactate dehydrogenase 2 | | 1 | 1 | 1 |

**Supplementary Table 4.** Genomic features related to protein degradation in *Microbacterium* strains

| Proteolytic system | | | | | | | |  |
| --- | --- | --- | --- | --- | --- | --- | --- | --- |
| Gene | Encoded protein | | *M. lacticum* DSM 20127 | | *Microbacterium* sp. 2C | | *M. lacticum* 18H | |
| **PROTEASE** |  | |  | |  | |  | |
| *clpS* | ATP-dependent Clp protease adapter protein ClpS | | 1 | | 1 | | 1 | |
| *clpC1* | ATP-dependent Clp protease ATP-binding subunit ClpC1 | | 1 | | 1 | | 1 | |
| *clpX* | ATP-dependent Clp protease ATP-binding subunit ClpX | | 1 | | 1 | | 1 | |
| *clpP1* | ATP-dependent Clp protease proteolytic subunit 1 | | 1 | | 1 | | 1 | |
| *clpP2* | ATP-dependent Clp protease proteolytic subunit 2 | | 1 | | 1 | | 1 | |
| *ftsH* | ATP-dependent zinc metalloprotease FtsH | | 3 | | 3 | | 3 | |
| *prtS* | Protease PrtS; zinc-metalloprotese | | 1 | | 1 | | 1 | |
| *yraA* | Putative cysteine protease YraA | | 0 | | 1 | | 0 | |
| *gluP* | Rhomboid protease GluP | | 1 | | 1 | | 1 | |
| *rip1* | Zinc metalloprotease Rip1 | | 1 | | 1 | | 1 | |
| **PEPTIDASE** |  | |  | |  | |  | |
| *apeB* | putative M18 family aminopeptidase 2 | | 1 | | 1 | | 1 | |
| *cpg2* | Carboxypeptidase G2 | | 0 | | 1 | | 0 | |
| *dap* | D-aminopeptidase | | 0 | | 2 | | 0 | |
| *dapb1* | Dipeptidyl aminopeptidase BI | | 1 | | 1 | | 1 | |
| *dcp* | Dipeptidyl carboxypeptidase | | 1 | | 1 | | 1 | |
| group_570 | Glycyl-glycine endopeptidase ALE-1 | | 1 | | 0 | | 1 | |
| group_6122 | Prolyl endopeptidase | | 0 | | 0 | | 1 | |
| *map-1* | Methionine aminopeptidase 1 | | 2 | | 2 | | 2 | |
| *mepM* | Murein DD-endopeptidase MepM | | 1 | | 0 | | 0 | |
| *pepA* | Cytosol aminopeptidase | | 1 | | 1 | | 1 | |
| *pepN* | Aminopeptidase N | | 2 | | 2 | | 3 | |
| *pepO* | Neutral endopeptidase | | 1 | | 1 | | 1 | |
| *pepPI* | Xaa-Pro aminopeptidase 1 | | 1 | | 1 | | 1 | |
| *pip* | Proline iminopeptidase | | 2 | | 2 | | 1 | |
| *prlC* | Oligopeptidase A | | 1 | | 1 | | 1 | |
| *yedK* | Putative SOS response-associated peptidase YedK | | 1 | | 1 | | 1 | |
| *ykfA* | putative murein peptide carboxypeptidase | | 0 | | 1 | | 0 | |
| *yodJ* | Putative carboxypeptidase YodJ | | 1 | | 1 | | 1 | |
| **PEPTIDE TRANSPORT** | |  | |  | |  | |  |
| *oppD* | Oligopeptide transport ATP-binding protein OppD | | 1 | | 1 | | 1 | |
| *oppF* | Oligopeptide transport ATP-binding protein OppF | | 1 | | 1 | | 1 | |
| *oppA* | Oligopeptide-binding protein OppA | | 2 | | 1 | | 1 | |

**Supplementary Table 5.** Other features (lipase, biofilm, heat and cold response) in *Microbacterium* strains

| Other features | | | | | |
| --- | --- | --- | --- | --- | --- |
| Gene | Encoded protein | *M. lacticum* DSM 20127 | *Microbacterium* sp. 2C | *M. lacticum* 18H |  |
| **LIPASE** |  |  |  |  |  |
|  | Monoacylglycerol lipase | 2 | 1 | 1 |  |
| *lip3* | Lipase 3 | 0 | 1 | 0 |  |
| **BIOFILM FEATURES** |  |  |  |  |  |
| *lptB* | Lipopolysaccharide export system ATP-binding protein LptB | 1 | 1 | 0 |  |
| *glf* | UDP-galactopyranose mutase | 1 | 1 | 2 |  |
| *epsF* | Type II secretion system protein F | 1 | 0 | 0 |  |
| *epsL* | putative sugar transferase EpsL | 0 | 1 | 0 |  |
| **COLD & HEAT RESPONSE** |  |  |  |  |  |
| *hslR* | Heat shock protein 15 | 1 | 1 | 1 |  |
|  | 18 kDa heat shock protein | 1 | 1 | 1 |  |
| *grpE* | Protein GrpE | 1 | 2 | 1 |  |
| *dnaK* | Chaperone protein DnaK | 1 | 1 | 1 |  |
| *dnaK2* | Chaperone protein dnaK2 | 1 | 0 | 1 |  |
| *dnaJ2* | Chaperone protein DnaJ 2 | 1 | 1 | 1 |  |
| *dnaJ* | Chaperone protein DnaJ | 1 | 0 | 1 |  |
| *cspA* | cold shock protein cspA | 1 | 1 | 1 |  |
| *clpB_2* | Chaperone protein ClpB | 2 | 2 | 2 |  |
